# Sensory Trick in a Patient with Cervical Dystonia: Insights from Magnetoencephalography

**DOI:** 10.3390/brainsci8040051

**Published:** 2018-03-22

**Authors:** Abhimanyu Mahajan, Andrew Zillgitt, Susan M. Bowyer, Christos Sidiropoulos

**Affiliations:** 1 Department of Neurology, Henry Ford Hospital, 2799 West Grand Boulevard, K-11, Detroit, MI 48202, USA; azillgi1@hfhs.org (A.Z.); sbowyer1@hfhs.org (S.M.B.); ccsidirop@gmail.com (C.S.); 2 Department of Neurology and Ophthalmology, Michigan State University, East Lansing, MI 48823, USA

**Keywords:** cervical dystonia, sensory trick, functional imaging, magnetoencephalography, botulinum toxin

## Abstract

Background: The proposed mechanisms for the sensory trick include peripheral sensory feedback to aid in correcting abnormal posture or movement. Case report: A 53-year-old woman with cervical dystonia underwent magnetoencephalography pre- and post-botulinum toxin injection and sensory trick, which was described as yawning. Study revealed connectivity between the left frontal and inferior frontal gyrus before yawning, which changed to the visual cortex and right middle frontal gyrus with yawning. Beta frequencies reduced and gamma frequencies increased after yawning. Discussion: The increase in gamma frequency bands may indicate increased GABAergic activity. Increase in connectivity in the right cerebellar region underscores the importance of cerebellum in pathogenesis of dystonia.

## 1. Introduction

Sensory tricks or ‘alleviating maneuvers’ are voluntary maneuvers that lessen the severity of abnormal movement or posture in people with dystonia. Up to 83% of cervical dystonia patients note partial to complete improvement with a sensory trick [1]. Sensory tricks may involve complex sensorimotor tasks such as singing or even yawning [2]. Although the exact mechanism by which a sensory trick is beneficial is not well known, proposed mechanisms have suggested peripheral sensory feedback to aid in correcting the abnormal posture or movement [3] and modulation of parietal lobe activity.

Deficits in visuospatial and executive pathways in people with dystonia have previously been described [3,4,5]. In addition, attenuation of abnormal muscle activity with a sensory trick has been associated with reduced activation of supplementary motor area and primary sensorimotor cortex [3]. Patients with adult-onset primary cervical dystonia with effective sensory tricks have been reported to have better visuotactile discrimination and shorter disease duration [4]. Magnetoencephalography (MEG) is a useful tool for analyzing brain connectivity in epilepsy, but has rarely been used in focal dystonias [6]. In this case study, we attempted to explore the mechanism of action of a sensory trick, using MEG for the first time, by comparing cerebral oscillations at the network level in a patient with cervical dystonia pre- and post-sensory trick, as well as pre- and post-administration of botulinum toxin.

## 2. Case Description

A previously healthy 53-year-old woman presented with an 8-month history of right torticollis and left laterocollis responsive to treatment with botulinum toxin injections. Her sensory trick consisted of alleviation of her symptoms upon yawning. MEG data were acquired in 10 min scans with eyes open, using a 148-channel whole-head magnetometer system (4D Neuroimaging, San Diego, CA, USA) inside a magnetically shielded room. A total of 4 resting-state MEG scans were collected, two right before the injection of botulinum toxin and another two 2 weeks after the injection of botulinum toxin. Pre-botulinum toxin injection scans were taken before and after yawning, and similarly after the injection of botulinum toxin.

The data were sampled at a rate of 508.6 Hz (DC to 100 Hz) and were then forward and backward bandpass filtered 3–50 Hz. An artifact filter, utilizing ICA, was used to remove heart signals observed in the MEG recordings. Then MEG Coherence Source Imaging (CSI) was performed to assess neuronal synchrony within different brain regions [6]. A Standard MRI was segmented, and the brain surface was represented by a cortical model of approximately 4000 dipoles, each having an x, y, and z orientation at each site. Sites were distributed to represent the same volume of cortical gray matter. This model was then morphed to fit the digitized head shape collected during the MEG acquisition.

Post-acquisition data processing was performed using MEG Tools, an open-source Matlab (The Mathworks Inc., Natick, MA, USA)-based software module for MEG brain imaging. MEG-CSI was quantified by applying a time frequency decomposition technique, the short-time Fourier transformation (sFFT). After transformation to a time frequency representation, the strengths of network interactions were estimated by calculation of coherence, a measure of synchrony between signals from different brain regions for each FFT frequency component. The 10 min of rest-state MEG data were prepared for source imaging by division into 80 segments, each containing 7.5 s of data of relatively uniform brain behavior [6]. For each of these data segments, signals from neuronal sources were isolated using an independent component analysis (ICA) spatiotemporal decomposition technique designed to extract signals from distinct compact sources that exhibit burst behavior and minimal temporal overlap with other active sources. These ICA signal components have MEG spatial magnetic field patterns corresponding to one or a few spatially distinct compact sources that are much easier to image accurately using a current-distribution source imaging technique (MR-FOCUSS) [7]. MR-FOCUSS only images amplitudes above 20% of the maximum amplituded threshold. Separate from the imaging algorithm, the cross-spectrum between ICA signals was calculated. In these cross-spectrum calculations, a sequence of FFT spectra were calculated using 0.5 s windows and 25% overlap with FFT amplitudes for 24 frequency bins 2 Hz in width between 3 and 50 Hz. The imaging results and the signal cross-spectrum were used to calculate the coherence between all pairings (~1400 locations in the brain) of active cortical locations within each of the 24 frequency bins. Finally, for each active source, the average coherence across frequencies and sources was calculated. In these MEG-CSI results, the localization of imaged brain activity is strongly dependent on the frequency bands with greatest power. Coherence analysis results were encoded as a color spectrum for values between 1 (entirely coherent) and 0 (no coherence) and overlaid on the patient’s MRI with the solutions restricted to to the gray matter. 

MEG-CSI is currently used in our hospital to identify hyperactive epileptogenic brain areas prior to surgery [8,9]. The variance between coherence from the patients pre- and post-sensory trick (yawn) and pre- and post-botulinum injections was assessed for statistical significance. 

### 2.1. Before the Injection of Botulinum Toxin

**Figure 1:**
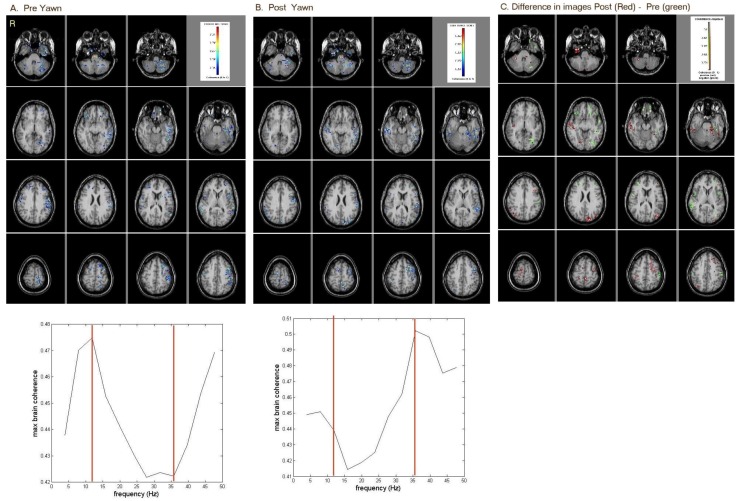
MEG-CSI results of resting-state brain-coherence activity (**A**) pre-sensory trick; (**B**) post-sensory trick; (**C**) subtraction of both images showing post-sensory trick (red) minus pre-sensory trick (green) differences. Prior to sensory trick, areas in the inferior frontal, left cerebellum and left parietal were active. After sensory trick, areas in the occipital and left temporal were more active.

Pre-sensory trick, areas of high coherence (0.47) peaking at 12 Hz were identified in the right and left inferior frontal lobe and left temporal region (Figure 1). Post-sensory trick, areas of high coherence (0.5) peaking at 36 Hz were present in multiple regions in both the right and left temporal lobes (Figure 1). Prior to the sensory trick, areas in the inferior frontal, left cerebellum and left parietal lobe were active (green in Figure 1). After the sensory trick areas, areas in the occipital and left temporal region were more active (red in Figure 1). Prior to the sensory trick, the coherence level at 36 Hz was 0.42, and at 12 Hz, it was 0.475. With sensory trick, the coherence level at 36 Hz increased to 0.5, and at 12 Hz, it decreased to 0.44. This indicates that the sensory trick increased Gamma (>25 Hz) activity while decreasing Alpha (8–12 Hz) activity.

### 2.2. After the Injection of Botulinum Toxin

**Figure 2:**
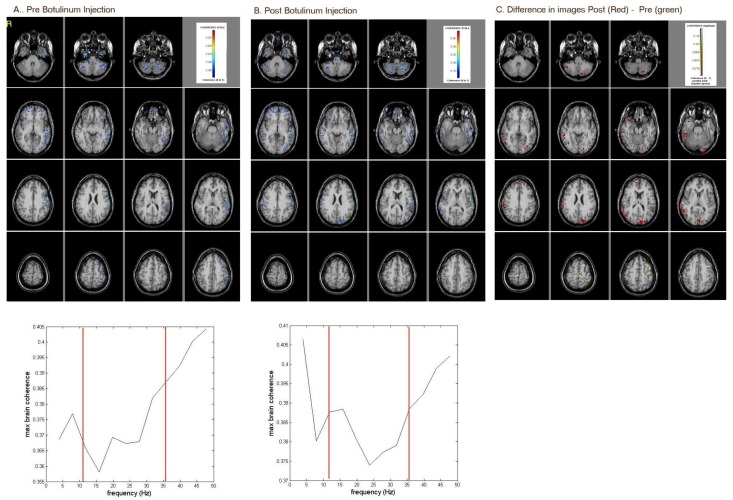
MEG-CSI results of resting-state brain-coherence activity (**A**) pre-botulinum injection; (**B**) post-botulinum injection; (**C**) subtraction of both images showing post-botulinum injection (red) minus pre-botulinum injection (green) differences. Prior to botulinum injection, areas in the superior partial and left parietal were active. After botulinum injection, areas in the occipital and right temporal were more active.

Areas of high coherence included the left temporal and parietal areas, as well as the right and left cerebellum (Figure 2). Post-botulinum toxin injection, areas of high coherence were noted in the left temporal region and in the right cerebellum (Figure 2). Prior to botulinum injection, areas in the superior partial and left parietal were active (green Figure 2). After botulinum injection, areas in the occipital, right cerebellum and right temporal were more active (red in Figure 2). Prior to botulinum toxin injection, the coherence level at 36 Hz was 0.39, and at 12 Hz, it was 0.37. With botulinum toxin, the coherence level at 36 Hz remained at 0.39, and at 12 Hz, it increased to 0.385. This indicates that the botulinum injection increased Alpha activity while maintaintng a constant Gamma activity.

## 3. Discussion

To the best of our knowledge, this is the first attempt to directly assess changes in functional connectivity with an effective sensory trick in a patient with cervical dystonia using MEG. In this study, we illustrated changes in coherence, connectivity, and frequency bands pre- and post-sensory trick and botulinum toxin treatment. The changes in coherence pre- and post-sensory trick and pre- and post-treatment with botulinum toxin underscore dynamic network changes that may share similar pathways, specifically within the visual cortex and frontal lobe. These results were corroborated with connectivity analysis, again illustrating changes within pathways involving the visual and frontal cortices. Furthermore, there were changes in frequency bands following both pre- and post-sensory trick and pre- and post-treatment with botulinum toxin. Specifically, the sensory trick resulted in reduced Alpha-band frequencies and an increase in Gamma-band frequency. Increased Gamma-band fluctuations in MEG have been shown to be positively correlated with GABA concentration in the same cortical region. Using a combination of MRI spectroscopy, MEG and visual psychophysics, Edden et al. showed that Gamma oscillation frequency positively correlated with GABA concentration in the primary visual cortex [10]. Using MEG, MRI spectroscopy and fMRI, Muthukumaraswamy et al. proved that gamma oscillation frequency is positively correlated with resting GABA concentration [11]. Similarly, our findings could support alterations within GABAergic pathways and a final common pathway in the sensory trick. Deficiency of intracortical inhibition is the keystone of pathophysiology in dystonia. These findings appear concordant with previous functional imaging studies in people with dystonia. Previous hypotheses have suggested that a sensory trick acts by enhancing pathways between the occipital and parietal lobes through proprioception [12]. Delnooz et al. demonstrated improved connectivity in people with primary cervical dystonia using functional MRI (fMRI) following treatment with botulinum toxin. In this study, there was improved connectivity between the sensorimotor and primary visual network after treatment with botulinum toxin [13].

It is well known that dystonia is a network disorder with clear involvement of the basal ganglia and cerebellum and their interaction [14]. The role of the cerebellum in dystonia is increasingly being studied, although it is not yet completely understood [15]. Our study implicates the cerebellum, temporal and parietal cortex, thereby adding to the literature on the cerebellum and sensorimotor control [16]. 

Our case study has some definite strengths. MEG measures the magnetic flux from electrical currents within the brain, and is therefore a direct measurement of neuronal activity. Furthermore, MEG has good spatial resolution (2–5 mm) and excellent temporal resolution (1 ms) [17,18]. These unique features make MEG an attractive tool for studying functional connectivity in a variety of neurological disorders [6,8,9]. 

MEG-CSI, using source-space localization, demonstrates excellent spatial resolution for cortical imaging [14]. Performing source-space coherence after current-distribution imaging negates current spread, thereby avoiding the relatively poor spatial resolution of sensor-space coherence. Our MEG lab sensor, a magnetometer-type system, was found to have better resolution for deeper structures.

We decided on a 3–50 Hz filter based on our experience with epilepsy pre-surgical mapping. While a 3Hz high-pass filter removes any breathing artifacts, a 50 Hz low-pass filter provides all the power that is in the data without any impact from the 60 Hz power lines. 

Overall, this case report utilized an underutilized approach, MEG-CSI, to study the effects of a sensory trick and botulinum toxin in a patient with cervical dystonia. Specifically, there were changes in coherence and connectivity within the visual and frontal cortices as well as reduced beta frequency bands and increased Gamma frequency bands pre- and post-trick and treatment. These findings support a dystonia network involving the visual and frontal cortices and potentially suggest a sensory trick and botulinum toxin similarly alter GABAergic pathways to create clinical improvement [10,11]. The case study nature of our report limits the ability to draw firm statistical conclusions, yet the widespread changes seen in connectivity and brain dynamics in the different conditions clearly indicate that this approach is suitable for further studies where quantitative statistics can better support new insight into the pathogenesis of dystonia at a network level.

To summarize, an effective sensory trick is associated with hyper-excitability in the parietal cortex and perhaps increased gamma frequency in this region. The parietal cortex, a center of sensorimotor integration, may play an integral role in reducing dystonia via amplification of Gamma frequencies. Botulinum toxin may have similar effects within the cortex and cerebellum, and these findings may indicate a common sensory pathway. Although these findings are intriguing and promising, replication in a larger patient cohort is necessary to verify their validity. 

## 4. Financial Disclosures of All Authors

Funding: The authors report no external funding for this case study.

Financial Disclosures: The authors have no relevant financial disclosures.

## 5. Ethics Statement

This study has been performed in accordance with the ethical standards laid down in the 1964 Declaration of Helsinki and its later amendments. 

The patient gave her informed consent prior to her inclusion in the study. No patient identifiable information is included in the text.
